# Validation of the German version of Infants and Toddlers Dermatology Quality of Life (InToDermQoL) Questionnaire

**DOI:** 10.1111/ddg.16008

**Published:** 2026-01-30

**Authors:** Juliane Traxler, Neuza da Silva Burger, Matthias Augustin, Hagen Ott, Sanna Hoffmann, Regina Fölster‐Holst, Maria Baumeister, Petra Staubach, Rachel Sommer

**Affiliations:** ^1^ German Center for Health Services Research in Dermatology (CVderm) Institute for Health Services Research in Dermatology and Nursing (IVDP) University Medical Center Hamburg‐Eppendorf (UKE) Hamburg Germany; ^2^ Paediatric Dermatology and Allergology Children's Hospital Auf der Bult Hannover Germany; ^3^ Clinic for Dermatology Venereology and Allergology University Medical Center Schleswig‐Holstein Kiel Germany; ^4^ Specialist Clinic Gaißach (Rehabilitation clinic for chronic diseases in children, teenagers and adults) Gaißach Germany; ^5^ Department of Dermatology University Medical Center Mainz Germany

**Keywords:** Atopic dermatitis, outcome measure, pediatric dermatology, psychometrics, quality of life, toddlers and infants

## Abstract

**Background and Objectives:**

Skin diseases can greatly impair quality of life (QoL) of pediatric patients and their families. The *Infants and Toddlers Dermatology Quality of Life questionnaire* (InToDermQoL) is the first skin‐generic instrument assessing QoL in children ≤ 4 years, as reported by their caregiver. This study aimed to psychometrically validate the German version of the InToDermQoL.

**Patients and Methods:**

The psychometric properties of the InToDermQoL were examined in a sample of 148 children with various skin diseases (age groups [n]: < 1 [44]; 1–2 [68]; 3–4 [36]).

**Results:**

Exploratory factor analysis suggested a unidimensional structure of the version for children < 1 year, and a two‐ and three‐factor structure for the two older age groups, respectively. Internal consistency was excellent (Cronbach's alpha, 0.904–0.922). Two‐week test–retest reliability was good, except in children aged 1–2 years (intraclass correlation coefficient [ICC], 0.430). The InToDermQoL demonstrated good convergent validity with other QoL questionnaires.

**Conclusions:**

The German InToDermQoL is a valid and reliable QoL measure in children ≤ 4 years with any skin disease. Application of the tool in research and clinical practice will contribute to a better understanding of the impact of pediatric dermatoses, and optimize care and support for affected families.

## INTRODUCTION

Skin diseases are highly prevalent in early childhood; atopic dermatitis (AD) alone has an estimated prevalence of 17% in infants aged 0–2 years and 13% in children aged 3–6 years in Germany.^1^ Importantly, pediatric dermatoses do not only cause physical symptoms but can substantially impair overall family functioning,^2^, ^3^ the child's psychosocial and physical development, self‐image, as well as health‐related quality of life (HRQoL).^4^, ^5^, ^6^, ^7^, ^8^ Hence, there is great need in clinical care, as well as in real‐world studies, to assess HRQoL of young patients with skin conditions. Understanding the burden of disease as well as the individual patients’ and caregivers’ needs and concerns, within a family‐centered approach, would enable healthcare providers to tailor treatment and optimize care. It would also contribute to a more person‐ and people‐centered care in these young patients and their families.^9^, ^10^, ^11^

However, few studies have focused on health‐related quality of life in young children and preschoolers with skin diseases, largely due to the limited availability of validated instruments. The development of such instruments is complicated by rapid developmental changes during the first years of life and by the need for proxy assessments provided by parents or other caregivers rather than direct patient questioning. In addition, while atopic dermatitis has a high prevalence, other skin conditions (e.g., psoriasis, pityriasis rosea, etc.) are rarer and make the development of disease‐specific questionnaires impracticable.

To address this gap, the *Infants and Toddlers Dermatology Quality of Life (InToDermQoL) questionnaire* was developed as a skin‐generic proxy‐measure of HRQoL,^12^ according to the methodology for cross‐cultural instrument development proposed by the European KIDSCREEN/DISABKIDS groups.^13^ The three age‐specific versions of the questionnaire, with 10, 12, and 15 items, respectively, were already psychometrically tested in some language versions, confirming high internal consistency, excellent test‐retest reliability and good convergent validity.^14^ Furthermore, the instrument's responsiveness and minimal clinically important difference were confirmed.^15^, ^16^

While Germany has participated in the pilot study phase of instrument development,^12^ the German version has not been previously validated. The current project aimed to psychometrically validate the German InToDermQoL in accordance with the international guidelines for assessment of measurement properties of PRO measures outlined by the U.S. Food and Drug Administration,^17^ for use in research and clinical practice.

## MATERIALS AND METHODS

This study had a cross‐sectional observational design and was conducted according to the guidelines for international field‐test of the InToDermQoL questionnaire and general recommendations for HRQoL measurement.^13^, ^18^, ^19^ Data collection took place between August 2021 and April 2024. The study was carried out in compliance with the Helsinki Declaration and approved by the ethics committee of the University Medical Center Hamburg‐Eppendorf (approval reference number: LPEK‐0304).

### Participants

Caregivers of an infant or toddler aged 0 to 4 years with any type of skin disease were invited to take part in this survey. Participants were recruited at six German dermatology centers and pediatric hospitals, and provided written informed consent prior to the study. Eligibility criteria were checked by the attending physician and comprised: being a caregiver of a child aged 0–4 years with a diagnosis of any type of skin disease; ability to consent autonomously and to speak and understand German.

### Outcome measures

Data were collected using pen and paper. The German versions of the following questionnaires were used.

### Clinician‐reported outcomes

*Global Clinical Assessment (GCA)*. The GCA is a one item measure of dermatosis severity assessed on a 5‐point Likert Scale ranging from 0 (not expressed) to 4 (very severe).

*SCORing Atopic Dermatitis (SCORAD)*.^19^ The SCORAD assesses the extent and severity of atopic dermatitis. A total score can be computed, which can range from 0 (minimal severity) to 103 (maximal severity). SCORAD scores < 25 are considered as mild atopic dermatitis, scores 25–59 as moderate atopic dermatitis, and scores ≥ 60 as severe atopic dermatitis. Psychometric properties of this instrument in infants and young children are good.^20^ This questionnaire was filled in only for patients with atopic dermatitis.

### Caregiver‐reported outcomes

*InToDermQoL*.^12^ The German pilot‐version of the InToDermQoL,^12^ was revised based on the final English version,^14^ and includes ten items for parents/caregivers of children under 1 year of age, two extra items for parents/caregivers of children aged 1 to 2 years, and another three additional items for parents/caregivers of children between 3 and 4 years of age. The recall period is one week, and the items are to be answered on a 4‐point scale, ranging from 0 (not at all) to 3 (very). The total score is computed by summing the score of each item, with a minimum score of zero and a maximum score of 30 for children < 1 year of age, 36 for 1–3‐year‐olds, and 45 for 3–4‐year‐olds, with higher scores indicating more HRQoL impairments. In addition, items can be grouped into two domains (symptoms and daily activities) in the versions for children under 3 years, and into three domains (symptoms, daily activities and relationships with the other people) for children over 3 years, although the factorial structure of the questionnaire has not been tested previously.

*Infants' Dermatitis Quality of Life Index (IDQOL)*.^21^ The IDQOL is a disease‐specific HRQoL questionnaire to be completed by parents of infants aged up to 4 years with atopic dermatitis. The questionnaire contains ten questions on symptoms and difficulties with mood, sleep, play, family activities, mealtimes, treatments, dressing, and bathing. Items are to be scored in a Likert scale ranging from 0 to 3; a total score can be computed from the sum of the ten items (0–30) with higher scores indicating more HRQoL impairments.

*Paediatric Quality of Life Inventory (PedsQL^TM^)*.^22^, ^23^ The PedsQL^TM^ was designed as a generic HRQoL instrument assessing parents’ perceptions of their child's generic HRQoL. The PedsQL^TM^ Infant Scales includes two age‐appropriate versions: one for 1–12 months‐old infants (36 items) and one for ages 13–24 months (45 items). Items are to be answered on a 5‐point Likert scale ranging from 0 (never a problem) to 4 (almost always a problem). Items are reverse‐scored and linearly transformed to a scale of 0–100, with higher scores indicating better HRQoL. The German version was shown to have good psychometric properties.^24^

### Procedure

The attending physician reported on sociodemographic (age, sex, height, weight) and clinical (skin disease, current treatment, comorbidities, affected body surface area [0–100%], disease severity [GCA, SCORAD]) information of the patient. At baseline (T0), caregivers reported sociodemographic information of both themselves (age, relationship to the child) and the child (age, sex, main caregiver), clinical information of the child (skin disease, age at diagnosis, allergies and hypersensitivities, current symptoms and medication, nutrition), and a set of questionnaires as described above. A quarter of the respondents was asked to fill in a shortened version of the questionnaire, including sociodemographic and clinical items, and the InToDermQoL, 14 days later (T1) and return it to the coordinating study center via envelope.

### Statistical analyses

The statistical analyses were conducted using the Statistical Package for the Social Sciences (SPSS v.27.0; IBM Corp., Armonk, NY). The significance level was established at a p‐value ≤ 0.05. Descriptive statistics of sociodemographic and clinical characteristics were obtained for the total sample and for the three age groups: children < 1 year of age; children between 1 and 3 years of age, and children between 3 and 4 years of age.

Item distribution characteristics including floor and ceiling effects, skewness and kurtosis were inspected. The *structural validity* of the German InToDermQoL was examined using exploratory factor analysis (EFA). Bartlett's test of sphericity and the KMO statistic,^25^ were used to ensure that the correlation matrix was not random. Common factor analysis was conducted using Principal Axis Factoring. As factors were assumed to be correlated, an oblimin rotation was employed. Pattern coefficients ≥ 0.3 were considered salient. Cross‐loadings (loadings salient on more than one factor) were rejected to achieve simple structure.^26^ Factors with at least three salient loadings, internal consistency of α ≥ 0.7, and being theoretically meaningful were considered adequate.

The *internal consistency* of the InToDermQoL questionnaire was assessed by using Cronbach's alpha coefficients. To determine two‐week *test‐retest reliability*, intraclass correlation coefficients (ICC) were calculated in an absolute agreement, two‐way mixed effects model.^27^ For both internal consistency and test‐retest reliability, coefficients ≥ 0.50, ≥ 0.75 and ≥ 0.90 were considered moderate, good, and excellent reliability, respectively. To assess whether the sample that filled in T1 was different from the total sample, independent t‐tests were run on the sociodemographic (age, sex) and clinical characteristics (SCORAD) of the child, the age of the caregiver filling in the questionnaire, and the InToDermQoL scores at T0. *Standard errors of measurement* for the three age versions were calculated as $\mathrm{SEM}=\frac{SD_{Difference}}{\surd 2}$.^28^, ^29^ *Minimal detectable change* (MDC) scores can be computed for individual subjects as well as for group comparisons, the former being informative for clinical practice to determine individual change, the latter being useful in interpreting mean scores of groups, e.g. in research settings.^29^ The MDC scores were calculated at the 95% confidence interval following the formula MDC_individual_ = SEM × 1.96 × √2 and $\mathrm{MD}C_{\mathrm{group}}=\frac{MDC_{\mathrm{individual}}}{\sqrt{n}}$.^29^, ^30^

Construct validity was determined using convergent validity and known‐groups validity. *Convergent validity* was examined with Pearson Correlation Coefficients between the skin‐generic InToDermQoL and previously validated generic instruments (PedsQL^TM^ Generic Core Module). For the subgroup of patients with atopic dermatitis, the InToDermQoL was also correlated with the disease‐specific IDQOL and SCORAD. *Known‐groups validity* was tested using ANOVAs comparing the InToDermQoL scores across GCA disease severity levels. The following a priori‐defined hypotheses were tested for each of the three age groups, of which at least 75% had to be met to confirm construct validity:

1. The InToDermQoL and PedsQL showed a moderate negative correlation (r = −0.30 to −0.49).^31^
2. The InToDermQoL and IDQOL showed a moderate to high positive correlation (r ≥ 0.30).^14^, ^31^
3. The InToDermQoL and SCORAD showed a small to moderate positive correlation (r = 0.10–0.49).^31^
4. Children with moderate to severe disease (GCA ≥ 2) had higher InToDermQoL scores than those with no or mild disease burden (GCA < 2).

## RESULTS

In total, 148 children (56.1% male) with a mean age of 1.85 years (standard deviation [SD] = 1.1) were included in the study. Demographic and clinical information of the included children and respondents is presented in Table 1.

**TABLE 1:** Demographic and clinical characteristics of included children and caregivers in the total sample and by age group (mean [M] ± SD).

		Total (n = 148)	< 1 year (n = 44)	1–2 years (n = 68)	3–4 years (n = 36)
***Child***					
Gender (%)	Female	65 (43.91)	21 (47.73)	23 (33.82)	21 (58.33)
	Male	83 (56.08)	23 (52.27)	45 (66.18)	15 (41.67)
Age (in years)		1.85 (± 1.12)	0.58 (± 0.54)	1.91 (± 0.56)	3.34 (± 0.47)
Age at onset (in years)		0.57 (± 0.59)	0.23 (± 0.16)	0.64 (± 0.55)	0.84 (± 0.79)
Duration of flare‐ups (in months)		2.28 (± 1.66)	2.66 (± 1.75)	2.23 (± 1.62)	2.00 (± 1.60)
Longest appearance‐free phase (in months)		2.38 (± 2.62)	0.83 (± 1.48)	2.56 (± 2.88)	2.99 (± 2.40)
Treatment (%)^*^	None	4 (16.2)	2 (4.5)	1 (1.5)	1 (2.8)
	Topical	130 (87.8)	34 (77.3)	61 (89.7)	35 (97.2)
	Systemic	18 (12.2)	7 (15.9)	11 (16.2)	0
	Other	17 (11.5)	10 (22.7)	5 (7.4)	2 (5.6)
GCA (%)	1	5 (3.4)	2 (4.5)	2 (2.9)	1 (2.8)
	2	52 (35.1)	15 (34.1)	28 (45.9)	11 (30.6)
	3	61 (41.3)	22 (50.0)	24 (39.3)	13 (36.1)
	4	18 (12.2)	5 (11.4)	7 (10.3)	6 (16.7)
BSA (in %)		18.06 (± 19.35)	15.37 (± 16.52)	19.30 (± 20.96)	19.37 (± 19.90)
SCORAD^**^		31.6 (± 16.00)	35.23 (± 13.60)	30.16 (± 17.52)	30.66 (± 15.33)
***Diagnosis***					
Non‐infectious	Atopic dermatitis		31	55	32
	Hemangioma		10	1	1
	Becker naevus		1		
	Lymphangioma		1		
	Neurofibromatosis		1		
	Seborrheic eczema		1		
	Irritant diaper dermatitis			2	1
	Ichthyosis vulgaris			1	1
	Alopecia areata			1	
	Cutaneous mastocytosis			1	
	Eczema infantum			1	
	Juvenile Xanthogranuloma			1	
	Keratosis pilaris			1	
	Milium			1	
	Onychodystrophy			1	
	Lichen vidal				1
	Psoriasis				1
	Tinea amiantacea				1
	Naevus		1		
Bacterial infection	Superinfection		2	1	
	Impetigo			1	
	Whitlow			1	
Parasite infestation	Scabies			2	
Fungal infection	Tinea capitis			1	
Viral infection	Herpes simplex			1	
	Urticaria exanthema				1
**Respondent**					
Mother (%)		131 (88.5)	37 (84.1)	62 (91.2)	32 (88.9)
Father (%)		14 (9.5)	6 (13.6)	4 (5.9)	4 (11.1)
Both parents (%)		1 (0.7)	1 (2.3)	–	–
Other (%)		1 (0.7)	–	1 (1.5)	–
Age (in years)		33.59 (± 5.48)	31.51 (± 4.45)	33.59 (± 5.39)	36.06 (± 5.84)

*Abbr*.: GCA, Global Clinical Assessment (1, very mild–mild; 2, mild–moderate; 3, moderate–severe; 4, severe–very severe); BSA, Body Surface Area; SCORAD, SCORing Atopic Dermatitis

* 14 children received more than one type of treatment

** n = 109

No ceiling effects were observed for any of the age groups; however, a floor effect was present in the youngest age group (27.3%) (Table 2). Skewness and kurtosis were within the acceptable range (± 1.5).^32^ Item‐total correlations were mostly above 0.50, with 1–2 items per age version showing only moderate correlations. As deletion of these items would not substantially increase Cronbach's alpha, these items were retained. Information on IDQOL and PedsQL^TM^ as well as detailed psychometric results of the InToDermQoL can be found in Online Supplements S1 and S2.

**TABLE 2:** Descriptive statistics of the InToDermQoL for the total sample and by age group at baseline and follow‐up (T1).

Age group	Item	Mean (SD)	Range	Skewness	Kurtosis	Floor effects	Ceiling effects	Cronbach's alpha	Item‐total correlation	Cronbach's alpha when item deleted
< 1 year	Full scale	8.57 (7.26)	0–26	0.197	−1.081	27.3%	0%	0.912		
	1	1.58 (1.36)							0.824	0.894
	2	1.05 (1.04)							0.765	0.897
	3	0.88 (0.94)							0.766	0.898
	4	1.20 (1.16)							0.887	0.888
	5	0.80 (0.97)							0.812	0.895
	6	0.53 (0.93)							0.472	0.914
	7	0.70 (0.97)							0.686	0.902
	8	0.38 (0.81)							0.416	0.916
	9	0.38 (0.74)							0.532	0.910
	10	0.50 (0.75)							0.627	0.906
1–2 years	Full scale	11.62 (8.31)	0–30	0.573	−0.574	5.9%	0%	0.904		
	1	1.87 (1.09)							0.623	0.896
	2	1.23 (1.09)							0.607	0.897
	3	1.15 (1.03)							0.719	0.891
	4	1.44 (1.16)							0.760	0.889
	5	1.03 (1.10)							0.836	0.885
	6	0.70 (0.97)							0.697	0.893
	7	0.59 (0.84)							0.591	0.898
	8	0.49 (0.87)							0.569	0.899
	9	0.36 (0.66)							0.601	0.899
	10	0.84 (1.04)							0.551	0.900
	11	0.97 (1.10)							0.635	0.896
	12	0.69 (1.08)							0.404	0.907

Age group	Item	Mean (SD)	Range	Skewness	Kurtosis	Floor effects	Ceiling effects	Cronbach's alpha	Item‐total correlation	Cronbach's alpha when item deleted
3–4 years	Full scale	15.03 (9.39)	2–38	0.941	0.001	2.8%	0%	0.922		
	1	2.26 (0.86)							0.544	0.920
	2	1.70 (0.91)							0.552	0.920
	3	1.44 (0.93)							0.532	0.921
	4	1.52 (0.89)							0.640	0.917
	5	1.07 (0.96)							0.767	0.913
	6	0.93 (1.21)							0.693	0.916
	7	0.70 (0.95)							0.823	0.911
	8	0.48 (0.85)							0.726	0.915
	9	0.63 (1.08)							0.760	0.913
	10	1.11 (1.01)							0.643	0.917
	11	1.00 (0.92)							0.688	0.916
	12	0.78 (1.05)							0.708	0.915
	13	0.44 (0.70)							0.582	0.919
	14	0.59 (0.69)							0.534	0.920
	15	0.15 (0.36)							0.380	0.924

**T1**										
< 1 year	Full scale	9.91 (6.47)	0–21	0.459	−0.733	9.1%	0%	0.876		
1–2 years	Full scale	6.72 (5.17)	0–18	0.580	−0.543	6.9%	0%	0.897		
3–4 years	Full scale	9.15 (7.96)	2–26	1.249	0.485	7.5%	0%	0.890		

### Structural validity

EFA suggested the version for infants (< 1 year) to be unidimensional, explaining 61% of the total variance with a Cronbach's alpha of 0.917. The version for 1–2‐year‐old children appeared to comprise two factors, “Symptoms” (5 items) and “Daily activities” (7 items). Lastly, a 3‐factor solution was deemed the most adequate structural representation of the version for 3–4‐year‐olds, with the factors “Daily activities” (6 items), “Symptoms” (4 items), and “Relationships with others” (5 items). Factor loadings and their characteristics for the two older age groups are presented in Tables 3 and 4, an extensive description of the EFA can be found in Online Supplement S2.

**TABLE 3:** Factor loadings, total variance explained, and Cronbach's alpha for the 2‐factor solution of the German version of the InToDermQoL in children aged 1–2 years (n = 68).

	Factor	
	1	2
Your child has bled due to their skin condition (from injured skin and/or mucous membranes) [Ihr Kind hat aufgrund seiner Hautkrankheit geblutet (von verletzter Haut und / oder Schleimhaut)]	**0.906**	
Your child had sleeping problems due to their skin condition [Ihr Kind hatte Schlafprobleme aufgrund seiner Hautkrankheit]	**0.805**	
Your child was in pain due to their skin condition [Ihr Kind hatte Schmerzen aufgrund seiner Hautkrankheit]	**0.801**	
Your child was itching and scratching because of their skin condition [Ihr Kind hatte Juckreiz und hat sich aufgrund seiner Hautkrankheit gekratzt]	**0.720**	
Your child had mood changes due to their skin condition [Ihr Kind hatte Stimmungsveränderungen aufgrund seiner Hautkrankheit]	**0.720**	
Your child had restrictions and limitations due to their skin condition (social, diet, physical activity and sports, pets, etc.) [Ihr Kind hatte Einschränkungen und Grenzen aufgrund seiner Hautkrankheit (sozial, Ernährung, körperliche Aktivität und Sport, Haustiere, usw.)]		**0.771**
Your child had problems eating due to their skin condition [Ihr Kind hatte Probleme beim Essen aufgrund seiner Hautkrankheit]		**0.708**
Your child was tired or exhausted due to their skin condition [Ihr Kind war müde oder erschöpft aufgrund seiner Hautkrankheit]		**0.662**
Your child had problems with physical activity (movements for infants; walking, crawling, etc. for toddlers) due to their skin condition [Ihr Kind hatte Probleme bei körperlicher Aktivität (Bewegungen für Säuglinge; Laufen, Krabbeln, usw. für Kleinkinder) aufgrund seiner Hautkrankheit]		**0.657**
Your child had problems bathing due to their skin condition [Ihr Kind hatte Probleme beim Baden aufgrund seiner Hautkrankheit]	0.331	**0.529**
Your child had problems with treatment (e.g. treatment at home, bandaging, skin care, etc.) due to their skin condition [Ihr Kind hatte Probleme mit der Behandlung (z. B. Behandlung zu Hause, Bandagieren, Hautpflege, usw.) aufgrund seiner Hautkrankheit]		**0.433**
Your child had problems getting dressed and undressed (irritation of the affected areas, pain) due to their skin condition [Ihr Kind hatte Probleme beim An‐ und Ausziehen (Reizung der betroffenen Stellen, Schmerzen) aufgrund seiner Hautkrankheit]	0.363	**0.364**
**Total variance explained (in %)**	49.9	13.8
**Cronbach's alpha**	0.910	0.843

Factor 1: “Symptoms”, Factor 2: “Daily activities”.

**TABLE 4:** Factor loadings, total variance explained, and Cronbach's alpha for the 3‐factor solution of the German version of the InToDermQoL in children aged 3–4 years (n = 36).

	Factor		
	1	2	3
Your child had problems with physical activity (movements for infants; walking, crawling, etc. for toddlers) due to their skin condition [Ihr Kind hatte Probleme bei körperlicher Aktivität (Bewegungen für Säuglinge; Laufen, Krabbeln, usw. für Kleinkinder) aufgrund seiner Hautkrankheit]	**0.924**		
Your child had problems eating due to their skin condition [Ihr Kind hatte Probleme beim Essen aufgrund seiner Hautkrankheit]	**0.812**		
Your child had restrictions and limitations due to their skin condition (social, diet, physical activity and sports, pets, etc.) [Ihr Kind hatte Einschränkungen und Grenzen aufgrund seiner Hautkrankheit (sozial, Ernährung, körperliche Aktivität und Sport, Haustiere, usw.)]	**0.807**		
Your child had problems bathing due to their skin condition [Ihr Kind hatte Probleme beim Baden aufgrund seiner Hautkrankheit]	**0.782**		
Your child had problems with treatment (e.g. treatment at home, bandaging, skin care, etc.) due to their skin condition [Ihr Kind hatte Probleme mit der Behandlung (z. B. Behandlung zu Hause, Bandagieren, Hautpflege, usw.) aufgrund seiner Hautkrankheit]	**0.656**		
Your child had problems getting dressed and undressed (irritation of the affected areas, pain) due to their skin condition [Ihr Kind hatte Probleme beim An‐ und Ausziehen (Reizung der betroffenen Stellen, Schmerzen) aufgrund seiner Hautkrankheit]	**0.595**	0.317	
Your child had sleeping problems due to their skin condition [Ihr Kind hatte Schlafprobleme aufgrund seiner Hautkrankheit]	0.395	**0.357**	
Your child was in pain due to their skin condition [Ihr Kind hatte Schmerzen aufgrund seiner Hautkrankheit]		**0.930**	
Your child has bled due to their skin condition (from injured skin and/or mucous membranes) [Ihr Kind hat aufgrund seiner Hautkrankheit geblutet (von verletzter Haut und / oder Schleimhaut)]		**0.844**	
Your child was itching and scratching because of their skin condition [Ihr Kind hatte Juckreiz und hat sich aufgrund seiner Hautkrankheit gekratzt]		**0.726**	
Other people's questions about your child's skin condition have upset your child [Die Fragen anderer Personen nach der Hautkrankheit Ihres Kindes haben Ihr Kind verärgert]			**0.752**
Your child was tired or exhausted due to their skin condition [Ihr Kind war müde oder erschöpft aufgrund seiner Hautkrankheit]			**0.748**
Your child was rejected by others because of his skin disease [Es gab Zurückweisungen von Anderen aufgrund seiner Hautkrankheit]			**0.733**
Your child had mood changes due to their skin condition [Ihr Kind hatte Stimmungsveränderungen aufgrund seiner Hautkrankheit]	0.339		**0.539**
Your child has felt different from their peers because of their skin condition [Ihr Kind hat sich aufgrund seiner Hautkrankheit anders gefühlt als Gleichaltrige]	0.391		**0.503**
**Total variance explained (in %)**	48.8	12.9	10.2
**Cronbach's alpha**	0.920	0.868	0.854

Factor 1: “Daily activities”, Factor 2: “Symptoms”, Factor 3: “Relationships with others”.

### Reliability

Cronbach's alpha of the InToDermQoL was 0.912 for the age group < 1 year, 0.904 for the age group 1–2 years, and 0.922 for the age group 3–4 years. McDonald's omega values were similar (< 1 year: ω = 0.921; 1–2 years: ω = 0.902; 3–4 years: ω = 0.922), indicating optimal internal consistency in all age groups. Fifty‐one families (34.5%) filled in the second questionnaire (T1) with a mean (M) of 17.4 days (SD = 10.3; range: 5–66) between the two measurements. Table 5 shows the test‐retest reliability (ICC), SEM and MDC per age group. All ICCs were > 0.70, indicating good test‐retest reliability across the span of two weeks, except for 1–2‐year‐old children (ICC_3,1_ = 0.430). SEMs ranged between 3.77 and 5.61 points; the MDC_individual_ scores ranged between 8.89 and 15.56 points.

**TABLE 5:** Intraclass correlation coefficients (ICC), standard error of measurement (SEM), and minimal detectable change (MDC) by age group.

Age group	n (retest)	ICC	95% CI	SEM	MDC_individual_	MDC_group_
< 1 year	11	0.791^**^	0.285–0.942	3.77	10.46	3.15
1–2 years	27	0.430^*^	−0.122–0.723	5.61	15.56	4.69
3–4 years	13	0.814^**^	−0.106–0.955	3.21	8.88	2.68

*Abbr*.: ICC, intraclass correlation coefficient; CI, confidence interval; SEM, standard error of measurement; MDC_individual_, minimal detectable change (individual); MDC_group_, minimal detectable change (group)

* p < 0.05

** p < 0.01.

### Construct validity

All correlations between the three InToDermQoL versions and IDQOL (all r > 0.7; for children with atopic dermatitis), SCORAD (0.1–0.57) and the respective age versions of PedsQL^TM^ (all r < –0.4), were found to be in the expected direction and magnitude (Table 6). Regarding known‐groups validity, higher InToDermQoL scores were found among the severely affected children compared to those with no or mild burden (GCA < 2) across age groups. However, the effect of GCA score on InToDermQoL was significant only among 1–2‐year‐old children (Table 7; for details see online supplementary Table S2). Altogether, ≥ 75% of the a priori hypotheses were confirmed, lending support to the construct validity of the InToDermQoL.

**TABLE 6:** Correlations between InToDermQoL scores, other health‐related quality of life measures, and disease severity.

		InToDermQoL age group			Hypotheses	Hypothesis confirmed?
		< 1 year	1–2 years	3–4 years		
PedsQL^TM^	0–12 months	−0.628^**^			InToDermQoL & PedsQL^TM^ show a moderate negative correlation	Yes
	13–24 months		−0.415^*^			Yes
	25–48 months		−0.666^**^	−0.404^*^		Yes
IDQOL		0.732^**^	0.772^**^	0.878^**^	InToDermQoL & IDQOL show a moderate to high positive correlation	Yes
SCORAD		0.109	0.565^**^	0.150	InToDermQoL & SCORAD show a small to moderate positive correlation	Yes

* p < 0.05 (two‐tailed)

** p < 0.01 (two‐tailed).

**TABLE 7:** Results of ANOVAs assessing known‐groups validity of the InToDermQoL.

Age group	InToDermQoL score		F‐value	p‐value	η_p_^2^	Hypothesis	Hypothesis confirmed?
	GCA < 2	GCA ≥ 2					
< 1 year	7.47 (± 6.97)	9.26 (± 7.49)	F(1,42) = 0.628	0.433	0.015	Children with moderate to severe disease (GCA ≥ 2) have higher InToDermQoL scores than those with no or mild burden (GCA < 2)	No
1–2 years	9.08 (± 7.02)	14.65 (± 8.80)	F(1,66) = 8.407	0.005^*^	0.113		Yes
3–4 years	13.06 (± 8.30)	16.79 (± 10.17)	F(1,34) = 1.432	0.240	0.040		No

* p < 0.01

## DISCUSSION

The primary objectives of this project were to revise the German pilot‐version of the InToDermQoL, the first skin‐generic HRQoL proxy‐instrument for young children below the age of 4 years, and to evaluate its psychometric properties across the three different age groups.

Overall, the analysis of the item characteristics and overall scale performance yielded positive results. The distribution of items across the different age groups was generally acceptable, with only one notable issue. A floor effect was observed in the youngest age group (children aged 0–12 months), reflecting that a significant portion (27.3%) of responses fell into the lowest quarter of the scale. This could suggest that the items are not sufficiently sensitive to detect variations in HRQoL for this age group. Nonetheless, no other significant concerns related to item distribution were observed.

Exploratory factor analysis yielded results largely in line with the domains proposed at instrument development:^12^ for the age group 1–2 years, two factors “Symptoms” and “Daily activities” are plausible, while the age group 3–4 years has a third factor “Relationships with others”. Deviating from the proposed two domains, the current data suggests the InToDermQoL_< 1 year_ to be unidimensional. However, in the first months, caregivers may find it particularly difficult to interpret their child's reactions to daily activities, making it challenging to differentiate between symptom‐related distress and distress from other causes. When the child's communication skills improve, it may become easier for the caregiver to tell these causes apart, such that two separate factors of the InToDermQoL emerge. As the child grows older, even more factors contribute to their HRQoL. At a very young age, the child's social interactions are predominantly those within the core family, who are likely to know about the skin disease, but as the child enters kindergarten and begins to interact with peers, they may be exposed to other people's reactions to their skin disease. In addition, children do not become self‐aware until around the age of 15 to 18 months,^33^ and start noticing differences in body representations from around 24 months on.^34^ Consequently, the visual and social aspects of skin disease begin to play a role in HRQoL as well. It is important to emphasize that these findings stem from EFA and need to be corroborated by CFA on larger datasets. Nevertheless, this is the first take on testing the factor structure of the InToDermQoL, which has generated important hypotheses for future validations.

The internal consistencies of the three age versions were excellent, and no significant improvements would be achieved by removing items from the questionnaire or scales. This indicates that the items are well‐selected, non‐redundant, and contribute meaningfully to the overall assessment of HRQoL in children with skin conditions. Test‐retest reliability over a two‐week period was good for the youngest and oldest age group. However, the reliability for the 1–2‐year‐old group was weak. This instability of QoL ratings might be explained by the rapid developmental changes in this age group in particular. The time interval of 2 weeks was selected to prevent recall bias, as is commonly recommended for the evaluation of test‐retest reliability.^35^, ^36^ However, young children often change quickly in terms of their behavior and communication skills, which also affects their parents’ perceptions. In the second year of life, important gross and fine motor skills (e.g., walking, eye‐hand coordination), as well as cognitive and first language abilities develop and refine.^37^ In addition, it is common in Germany to enter Kindergarten around the age of 12 months, with the end of parental leave. This broadening of contexts may modulate and prompt rapid changes in both child behavior and parental perceptions through social comparison with other children and the sharing of experiences among caregivers. Thus, further studies are necessary to confirm whether the weak test‐retest reliability in this specific age group is a psychometric weakness of the questionnaire or reflects real changes in the construct being measured.

Convergent validity analyses showed correlations of expected direction and magnitude with general HRQoL (PedsQL^TM^), and with atopic dermatitis‐related QoL (IDQOL) and disease severity (SCORAD) among children with atopic dermatitis. This suggests that the InToDermQoL effectively captures the construct “skin disease‐related QoL” in young children as reported by their caregiver. A particularly noteworthy finding was the strong positive correlation between InToDermQoL scores for 1–2‐year‐olds and disease severity as measured by SCORAD. This could imply that disease severity plays a particularly important role for HRQoL in this age group. This may also help explain the relatively lower test‐retest reliability observed for 1–2‐year‐olds, as the changes in disease severity may lead to greater fluctuations in caregiver‐reported HRQoL. Concerning known‐groups validity, the scale did not perform well in differentiating between mildly and moderately to severely affected cases, which could be explained by the relatively small sample size. Overall, construct validity was confirmed nonetheless.

While the results of this study are promising, several limitations must be acknowledged. First, as anticipated based on previous studies,^14^, ^15^ and the generally high prevalence of atopic dermatitis, the majority of children included in the study were diagnosed with this condition. This limits the generalizability of the findings to children with other dermatological conditions. Future studies should aim to recruit a more diverse sample of children with various skin diseases to ensure the instrument's broader applicability. Second, most respondents were mothers. Although prior research has not shown significant differences in proxy‐ratings of children's HRQoL between mothers and fathers using the IDQOL,^38^, ^39^ the interrater‐reliability of the InToDermQoL remains to be tested. Future studies should aim to collect ratings from both parents and other caregivers where possible to assess whether different caregivers provide consistent assessments of children's HRQoL. In addition, a future intervention study is needed to establish the minimal important difference (MID) of the German version of the InToDermQoL and test its responsiveness to changes in children's health status, which is critical for its use in both clinical practice and research.

The InToDermQoL is the first skin‐generic HRQoL instrument for children under the age of 4 years, now available in German language. In line with other language versions, its German version demonstrated good reliability and validity across all age groups in the present study. The instrument holds significant potential for use in both research and clinical settings, as it provides valuable insights into the impact of skin diseases on the HRQoL of young children and their families. Thus, the instrument has the potential to contribute to a more people‐centered approach of health care as emphasized by WHO,^11^ and others.^40^ In this sense, enhancing our understanding of how skin conditions affect this vulnerable population of infants and toddlers can ultimately contribute to improved medical care and better long‐term outcomes for children with dermatological conditions.

## FUNDING INFORMATION

This study was funded by Eli Lilly Germany.

## CONFLICT OF INTEREST STATEMENT

M.A. has served as a consultant, lecturer, and researcher and/or has received institutional research grants from companies manufacturing drugs for skin diseases in children, including AbbVie, Almirall, Amgen, Bayer, Beiersdorf, Eli Lilly, Galderma, Incyte, Janssen, LEO, L'Oréal, Novartis, Pfizer, Regeneron, Sanofi, and Viatris. P.S. has served as a consultant, lecturer, and researcher and/or has received institutional research grants from companies manufacturing drugs for skin diseases in children, including AbbVie, Almirall, Amgen, Beiersdorf, Biocryst, BMS, Celltrion, CSL Behring, Eli Lilly, Galderma, Incyte, Janssen, LEO Pharma, Regeneron, Sanofi, L'Oréal, Klinge, Pierre Fabre, Novartis, Pfizer, Stadapharma, Takeda, Unna Academy, and UCB Pharma. R.F.‐H. has served as a consultant and speaker for Allergika, Infectopharm, La Roche‐Posay, LEO, Novartis, Pierre Fabre, Regeneron, Sanofi, and Viatris. J.T., N.d.S.B., H.O., S.H., M.B., and R.S. declare no conflict of interest.

## Supporting information



Supplementary information

Supplementary information
