# Supplementary material for: Validation of the German version of Infants and Toddlers Dermatology Quality of Life (InToDermQoL) Questionnaire
Source: J Dtsch Dermatol Ges. 2026 Jan 30;24(9):1251–62. doi: 10.1111/ddg.16008 (PMC13551280; doi:10.1111/ddg.16008)
Supplement: Supplementary file 1 [file DDG-24-1251-s001.docx]

**Supplement 1**

**Table S1.** Descriptives and internal consistencies (Cronbach’s alpha) of IDQOL and PedsQL^TM^ for the total sample and differences across age groups.

| **Questionnaire** | **Scale** | **Age group** | | **M (SD)** | **Range** | **Between-group differences** | **Cronbach’s alpha** |
| --- | --- | --- | --- | --- | --- | --- | --- |
| IDQOL |  | total | | 8.58 (5.89) | 0 – 28 |  | 0.880 |
|  |  | < 1 year | | 8.36 (5.09) | 0 – 19 | F (2.221, 25.16) = 0.063, p = 0.939 |  |
|  |  | 1 – 2 years | | 8.56 (6.22) | 1 – 28 |  |  |
|  |  | 3 – 4 years | | 8.86 (6.02) | 1 – 24 |  |  |
| PedsQL^TM^ | total | < 1 year | | 79.74 (10.66) | 54.85 – 99.31 |  | 0.877 |
|  | physical |  |  | 79.52 (17.49) | 41.67 – 100 |  |  |
|  | symptoms |  |  | 83.73 (14.62) | 17.50 – 100 |  |  |
|  | emotional |  |  | 72.83 (14.02) | 45.83 – 100 |  |  |
|  | social |  |  | 89.18 (14.99) | 50 – 100 |  |  |
|  | cognitive |  |  | 83.33 (18.38) | 37.50 – 100 |  |  |
|  | total | 1 – 2 years* | 13 – 24 | 85.10 (7.54) | 70 – 100 |  | 0.872 |
|  |  |  | 25 – 36 | 84.45 (8.67) | 58.75 – 100 |  |  |
|  | physical |  | 13 – 24 | 84.47 (13.57) | 61.11 – 100 |  |  |
|  |  |  | 25 – 36 | 87.42 (10.49) | 56.25 – 100 |  |  |
|  | symptoms |  | 13 – 24 | 92.15 (5.89) | 82.50 – 100 |  |  |
|  | emotional |  | 13 – 24 | 73.56 (13.27) | 54.17 – 100 |  |  |
|  |  |  | 25 – 36 | 71.35 (14.09) | 45 – 100 |  |  |
|  | social |  | 13 – 24 | 93.20 (9.23) | 65 – 100 |  |  |
|  |  |  | 25 – 36 | 91.45 (10.77) | 60 – 100 |  |  |
|  | cognitive |  | 13 – 24 | 89.31 (13.94) | 50 – 100 |  |  |
|  | daycare |  | 25 – 36 | 88.11 (16.03) | 41.67 – 100 |  |  |
|  | total | 3 – 4 years | | 85.13 (13.51) | 51.19 – 100 |  | 0.837 |
|  | physical |  |  | 88.28 (16.12) | 40.63 – 100 |  |  |
|  | emotional |  |  | 77.92 (17.66) | 35 – 100 |  |  |
|  | social |  |  | 91 (13.66) | 55 – 100 |  |  |
|  | school |  |  | 85.35 (18.75) | 50 – 100 |  |  |

*PedsQL^TM^ uses different age ranges than InToDermQoL, such that parents of children in the age group 1 – 2 years filled in either the PedsQL^TM^ version for children aged 12 – 24 months or 25 – 48 months, depending on the exact age of their child.
