# Supplementary material for: Validation of the German version of Infants and Toddlers Dermatology Quality of Life (InToDermQoL) Questionnaire
Source: J Dtsch Dermatol Ges. 2026 Jan 30;24(9):1251–62. doi: 10.1111/ddg.16008 (PMC13551280; doi:10.1111/ddg.16008)
Supplement: Supplementary file 2 [file DDG-24-1251-s002.docx]

**Supplement 2**

*Detailed results*

*Structural validity.* Bartlett’s test of sphericity indicated that the correlation matrix was not random (< 1 year: *χ^2^* (45) = 290.95, *p* < 0.001; 1 – 2 years: *χ^2^* (66) = 453.31, *p* < 0.001; 3 – 4 years: *χ^2^* (105) = 286.69, *p* < 0.001). The KMO statistic was above the recommended threshold of 0.6^25^ (< 1 year: 0.807; 1 – 2 years: 0.823; 3 – 4 years: 0.690). Therefore, it was determined that data was appropriate for factor analysis.

In the youngest age group, the scree plot suggested retention of two factors. Eight items loaded on Factor 1 and two items loaded on Factor 2, one of which cross-loaded on Factor 1 (0.32). Factor 1 accounted for 56.7 % of the total variance and had a Cronbach’s alpha of 0.921; Factor 2 accounted for 13.2 % of the total variance and had a Cronbach’s alpha of 0.763. The two factors correlated at 0.324, showing a relatively similar pattern and structure matrix. Alternatively, a 3-factor solution was explored, in which six items loaded on Factor 1, of which two also cross-loaded on Factor 3 (both > 0.34), and two items each loaded on Factors 2 and 3. Factor 1 accounted for 56.7 % of the total variance and had a Cronbach’s alpha of 0.929. Factor 2 accounted for 13.2 % of the total variance and had a Cronbach’s alpha of 0.763. Factor 3 accounted for 9.5 % of the total variance and had a Cronbach’s alpha of 0.729. Factors 1 and 2 correlated at 0.484, Factors 1 and 3 correlated at 0.550, and Factors 2 and 3 correlated at 0.209, reflecting some divergence between pattern and structure matrix. None of these solutions met all four criteria, as in both cases at least one factor was loaded by less than three variables. A one-factor solution with all 10 items did not meet the criterion of explaining > 60 % of the total variance. However, when deleting item 8 (“eating problems”), which was the only one not loading on Factor 1 in the 2-factor solution, this criterion was met. The unidimensional scale with nine items explained 60.97 % of the total variance and had a Cronbach’s alpha of 0.917. While two domains were initially proposed for this age version,^12^ a unidimensional structure is theoretically plausible: as infants have very little autonomy and strongly limited abilities to communicate their needs, it may be particularly challenging for caregivers ascribe crying, withdrawal from touch, etc., to the skin disease versus other circumstances, i.e. to differentiate between skin disease-related reactions and more general moods. This hypothesis needs to be cross-tested in another sample using CFA.

For the age group 1 – 2 years, the scree plot suggested retention of two factors. Five items loaded on Factor 1 and seven items loaded on Factor 2, two of which cross-loaded on Factor 1 (both > 0.33). Factor 1 accounted for 49.9 % of the total variance and had a Cronbach’s alpha of 0.910; Factor 2 accounted for 13.8 % of the total variance and had a Cronbach’s alpha of 0.843. The two factors correlated at 0.497, showing a largely similar pattern and structure matrix. Factor 1 was interpreted as “Symptoms”, whereas Factor 2 was coined “Daily activities” in alignment with what has been proposed at instrument development.^12^ This two-factor solution (see Table 3) was deemed the most adequate structural representation of the InToDermQoL version for children aged 1 – 2 years.

For the age group 3 – 4 years, the scree plot suggested retention of three factors. Seven items loaded on Factor 1, two of which cross-loaded on Factor 2 (> 0.31), three items loaded on Factor 2, and five items loaded on Factor 3, two of which cross-loaded on Factor 1 (> 0.33). Factor 1 accounted for 48.8 % of the total variance and had a Cronbach’s alpha of 0.919. Factor 2 accounted for 12.9 % of the total variance and had a Cronbach’s alpha of 0.888. Factor 3 accounted for 10.2 % of the total variance and had a Cronbach’s alpha of 0.854. Factors 1 and 2 correlated at 0.367, Factors 1 and 3 correlated at 0.497, and Factors 2 and 3 correlated at 0.317, reflecting relatively similar pattern and structure matrices. In an alternative two-factor solution, twelve items loaded on Factor 1, one of which cross-loaded on the other factor (0.34), and three items loaded on Factor 2. Factors 1 and 2 showed Cronbach’s values of α = 0.921 and α = 0.888, respectively. The two factors correlated at 0.439, showing a largely similar pattern and structure matrix. The three-factor solution (see Table 4) was deemed the most adequate structural representation of the InToDermQoL version for children aged 3 – 4 years. Aligning with the proposed domains,^12^ Factor 1 was interpreted as “Daily activities”, Factor 2 was termed “Symptoms”, and Factor 3 was interpreted as “Relationships with others”. Given that item 4 (“sleeping problems”) cross-loads on Factor 2 “Symptoms” and has a better theoretical fit with this factor (see age group 1 – 2 years), item 4 was assigned to Factor 2 in the final model. Hence, Factor 1 consists of six items with a Cronbach’s alpha of 0.920; Factor 2 consists of four items with a Cronbach’s alpha of 0.868, while Factor 3 remains unchanged.

*Reliability*. Fifty-one families (34.46 %) filled in the second questionnaire (T1) with *M* = 17.40 days (*SD* = 10.26; range: 5 – 66) between the two measurements. In two cases, the T0 and T1 questionnaires were not filled in by the same parents. There were no significant differences between the sociodemographic and clinical variables of the child, or the age of the responding caregiver between those who filled in the T1 questionnaire compared to those who did not (all *p* > 0.1). Importantly, in the youngest age group, the InToDermQoL score at T0 was significantly larger among the infants whose parents filled in T1 (*M* = 12.45, *SD* = 6.91) compared to those who did not (*M* = 7.27, *SD* = 7.00; *t (42)* = 2.133, *p* = 0.039, 95 % CI [0.038, 1.439]). For the two older age groups, the InToDermQoL scores did not differ between the completers and non-completers (both *p* < 0.4).

SEms ranged between 3.77 and 5.61 points, indicating that the ‘true’ score of a subject falls between -3.77/5.61 and +3.77/5.61 points from the observed score with 68 % confidence (± 1 SEm), and between -7.54/11.22 and +7.54/11.22 points of the observed score with 95 % confidence (± 2 SEm). The MDC_individual_ scores ranged between 8.88 and 15.56 points. This reflects that a child’s score on the InToDermQoL would have to change with at least 8.88 points (on a scale of 0 to 30) for children < 1 year, 10.46 points (on a scale of 0 to 36) for 1 – 2-year-olds, and 15.56 points (on a scale of 0 to 45) for 3 – 4-year-olds, so that the observed change can be considered a true change in HRQoL, rather than a potential result of measurement error.

*Known-groups validity*. Table S2 shows the relevant characteristics of the subgroups, i.e. at different disease severity levels as assessed by GCA.

**Table S2**. Demographics and clinical characteristics [M (± SD)] of included children across age groups by disease severity (GCA).

|  |  | **< 1 year** | **1 – 2 years** | **3 – 4 years** |
| --- | --- | --- | --- | --- |
| **GCA < 2** |  | **(n = 17)** | **(n = 37)** | **(n = 17)** |
| Gender (%) | female | 8 | 13 | 8 |
|  | male | 9 | 24 | 9 |
| Age (in years) | | 0.57 (± 0.27) | 1.87 (± 0.57) | 3.42 (± 0.50) |
| Age of onset (in years) | | 0.23 (± 0.19) | 0.66 (± 0.51) | 0.76 (± 0.68) |
| Treatment (%)* | none | 1 (5.9) | 1 (2.8) | 1 (5.9) |
|  | topical | 10 (58.8) | 33 (91.7) | 16 (94.1) |
|  | systemic | - | 5 (13.9) | - |
|  | other | 7 (41.2) | 2 (5.6) | 1 (5.9) |
| BSA (in %) | | 9.15 (± 11.29) | 12.74 (± 17.49) | 7.79 (± 6.18) |
| SCORAD‡ | | 25.96 (± 6.33) | 22.99 (± 13.27) | 20.15 (± 10.44) |
| **GCA ≥ 2** | | **(n = 27)** | **(n = 31)** | **(n = 19)** |
| Gender (%) | female | 13 | 10 | 13 |
|  | male | 14 | 21 | 6 |
| Age (in years) | | 0.59 (± 0.28) | 1.97 (± 0.54) | 3.28 (± 0.45) |
| Age of onset (in years) | | 0.23 (± 0.14) | 0.62 (± 0.61) | 0.93 (± 0.90) |
| Treatment (%)* | none | 1 (3.7) | 30 (100) | - |
|  | topical | 24 (88.9) | 28 (93.3) | 19 (100) |
|  | systemic | 7 (25.9) | 6 (20) | - |
|  | other | 3 (11.1) | 3 (10) | 1 (5.3) |
| BSA (in %) | | 19.28 (± 18.21) | 26.06 (± 22.33) | 26.68 (± 22.16) |
| SCORAD‡ | | 39.62 (± 14.01) | 38.24 (± 18.42) | 39.86 (± 12.91) |

GCA = Global Clinical Assessment (< 2: no burden – mild, ≥ 2: moderate – very severe); BSA = Body Surface Area; SCORAD = SCORing Atopic Dermatitis; *some children received more than one type of treatment. ‡only filled in for children with atopic dermatitis
